# Positionspapier Schlaganfallnachsorge der Deutschen Schlaganfall-Gesellschaft – Teil 3: Strukturelle Konzepte für zukünftige Versorgungsformen der Schlaganfallnachsorge

**DOI:** 10.1007/s00115-021-01230-w

**Published:** 2021-12-21

**Authors:** Christopher J. Schwarzbach, Dominik Michalski, Markus Wagner, Tobias Winkler, Stephen Kaendler, Matthias Elstner, Andrea Dreßing, Joseph Claßen, Andreas Meisel, Armin Grau, Mitglieder Kommission Nachsorge der Deutschen Schlaganfall-Gesellschaft

**Affiliations:** 1 Neurologische Klinik, Klinikum der Stadt Ludwigshafen am Rhein, Bremserstr. 79, 67063 Ludwigshafen, Deutschland; 2 Klinik und Poliklinik für Neurologie, Universitätsklinikum Leipzig, Leipzig, Deutschland; 3 Stiftung Deutsche Schlaganfall-Hilfe, Gütersloh, Deutschland; 4 Klinik für Neurologie, kbo-Inn-Salzach-Klinikum, Wasserburg am Inn, Deutschland; 5 Praxis Kaendler & Wurtz, Offenbach, Deutschland; 6 Klinik für Neurologie, Klinikum Ansbach, Ansbach, Deutschland; 7 Klinik für Neurologie und Neurophysiologie, Universitätsklinikum Freiburg, Freiburg, Deutschland; 8 Centrum für Schlaganfallforschung Berlin und Klinik und Hochschulambulanz für Neurologie, Charité – Universitätsmedizin Berlin, corporate member of Freie Universität Berlin, Humboldt-Universität zu Berlin, and Berlin Institute of Health (BIH), Berlin, Deutschland

**Keywords:** Schlaganfall, Strukturelle Versorgungskonzepte, Anforderungen, Finanzierung, Perspektiven, Stroke, Structural care models, Requirements, Financing, Perspectives

## Abstract

**Hintergrund:**

Ungeachtet der großen gesamtgesellschaftlichen Bedeutung des Schlaganfalls sowie der Fortschritte in der Akutversorgung und Rehabilitation konnten bisher keine flächendeckenden Versorgungsstrukturen zur strukturierten ambulanten Nachsorge in Deutschland etabliert werden.

**Ziel der Arbeit und Methode:**

Vor dem Hintergrund der bestehenden Versorgungslücken wurde im Mai 2020 die Kommission Schlaganfallnachsorge der Deutschen Schlaganfall-Gesellschaft (DSG) gegründet. Das Positionspapier diskutiert strukturelle Konzepte für zukünftige Versorgungsformen der Schlaganfallnachsorge.

**Ergebnisse und Diskussion:**

Eine neurologische Betreuung sollte zentraler Bestandteil einer multidisziplinären, interprofessionellen und sektorenübergreifenden Behandlungsplanung sein. Strukturelle Konzepte zur Schlaganfallnachsorge müssen sowohl regionale Strukturunterschiede als auch den Aspekt der Qualitätssicherung berücksichtigen. Zertifizierungsprozesse und die angemessene Finanzierung von Nachsorgeregistern auf Landes- und Bundesebene können den Weg hierhin mittelfristig ebnen. Das Angebot zur strukturierten Schlaganfallnachsorge sollte allen Subgruppen von Schlaganfallpatienten offenstehen. Auch innovative Technologien können einen wichtigen Beitrag zur Schlaganfallnachsorge leisten. Die Einführung und Umsetzung einer strukturierten Schlaganfallnachsorge bedarf in jedem Fall einer ausreichenden Finanzierung und eigener finanzieller Anreize für die Leistungsträger. Die Vor- und Nachteile der spezifischen Versorgungs- und Finanzierungsmodelle müssen dabei kritisch gegeneinander abgewogen werden. Die Diskussion neuer Versorgungsformen zur Schlaganfallnachsorge erfährt gegenwärtig ein neues Momentum und eröffnet Perspektiven für eine Verbesserung der aktuell noch unzureichenden Versorgungslösung.

## Abstract (en)

**Background:**

Irrespective of the great impact stroke exerts on the society as a whole and far-reaching advances in acute treatment and rehabilitation of stroke, so far outpatient services for post-stroke care have not been established on a national level in Germany.

**Objective and methods:**

Against the background of this contemporary lack of care, in May 2020 the German Stroke Society (DSG) established the stroke aftercare commission. This position paper discusses structural models of future services addressing outpatient post-stroke care.

**Results and discussion:**

The specialized care by a neurologist should be central to a multidisciplinary, interprofessional and transsectoral treatment. Structural concepts of post-stroke care must take regional differences but also effective strategies for quality control into account. Certification processes and appropriate financing of follow-up registries at state and federal levels may pave the way for improvement over the medium term. Structured outpatient post-stroke care services should be open to all subgroups of stroke patients. Additionally, innovative technologies can make an important contribution to post-stroke care; however, the implementation of specialized services demands adequate funding as well as separate financial incentives for the providers. The solution must carefully balance the advantages and disadvantages of the specific care and financing models. Currently the discussion of new models of post-stroke care is gaining new momentum, which opens up perspectives for the advancement of the otherwise still insufficient contemporary care structures.

## Einleitung

Mit einer Lebenszeitprävalenz von 1,76 Mio. Betroffenen im Alter über 18 Jahren stellt der Schlaganfall eine der führenden gesundheitspolitischen Herausforderungen in Deutschland dar [1]. Auch gesundheitsökonomisch kommt dem Schlaganfall eine herausragende Bedeutung zu. Allein im Jahr 2015 entstanden durch zerebrovaskuläre Erkrankungen Behandlungskosten in Höhe von 4,7 Mrd. € [2]. Folgekosten sind hierbei noch nicht berücksichtigt und noch einmal höher einzuschätzen [3]. Neben der Rezidivprophylaxe erfordert insbesondere das weite Feld nichtvaskulärer Komplikationen sowie das Management persistierender neurologischer Defizite eine langfristige und multidisziplinäre Versorgung der Betroffenen [4].

Durch das bereits Mitte der 1990er-Jahre etablierte Zertifizierungsverfahren der Stroke-Units werden qualitative Mindeststandards in der Akutversorgung definiert und sichergestellt [5]. Ungeachtet der gesamtgesellschaftlichen Bedeutung und Fortschritte im Rahmen der Akutversorgung und Rehabilitation nach ischämischem Schlaganfall konnten bisher keine flächendeckenden Versorgungsstrukturen zur strukturierten ambulanten Schlaganfallnachsorge etabliert und somit keine gleichbleibend hohe Versorgungsqualität im chronischen Krankheitsverlauf sichergestellt werden [6]. Dieser Missstand bleibt gerade vor dem Hintergrund des bereits seit 2003 etablierten Disease-Management-Programms (DMP) für die koronare Herzerkrankung unter Berücksichtigung des vergleichbaren Risikoprofils unverständlich. Bereits im Jahr 2004 wurde die Notwendigkeit zur Etablierung von Strukturen zur umfassenden Schlaganfallnachsorge durch die Weltgesundheitsorganisation benannt und zuletzt durch die European Stroke Organisation (ESO) in Kooperation mit der Stroke Alliance for Europe (SAFE) in den „Action Plan for Stroke in Europe 2018–2030“ (SAP-E) in Form der Domäne „Life after stroke“ aufgenommen [7, 8]. Die Implementierung einer verbesserten strukturierten Schlaganfallnachsorge in die Regelversorgung ist in Deutschland jedoch ausstehend.

## Ziel der Arbeit und Methode

Vor dem Hintergrund der bestehenden Versorgungslücken wurde im Mai 2020 die Kommission Schlaganfallnachsorge der Deutschen Schlaganfall-Gesellschaft (DSG) gegründet. Hintergrund dieser Initiative ist die Auffassung, dass der Schlaganfall eine komplexe und vor allem chronische Erkrankung ist, welche eine langfristige und strukturierte Nachbehandlung erfordert. Diese bezieht sowohl Aspekte der Rezidivprophylaxe als auch des komplexen Managements nichtvaskulärer Komplikationen (Depression, Stürze, Fatigue etc.) und der sozialen Folgen unter dem Fokus des teilhabeorientierten Erhalts und der Verbesserung der Lebensqualität mit ein [9]. Ziel der Kommissionsarbeit ist es, Konzepte zur Verbesserung der Versorgungsrealität von Schlaganfallpatienten zu erarbeiten und deren Überführung in die Regelversorgung zu unterstützen. Die Aufarbeitung erfolgt in drei Arbeitsgruppen zu den Domänen:

1. aktuelle Versorgungsrealität und bestehende Versorgungsdefizite,
2. inhaltliches Konzept zur Diagnostik, zur Therapie und zum Umfang der Nachsorge,
3. strukturelles Konzept zukünftiger Versorgungsformen.

Ziel dieses Positionspapiers ist es, strukturelle Konzepte für zukünftige Versorgungsformen der Schlaganfallnachsorge zu diskutieren und dadurch Impulse für eine Umsetzung in die Regelversorgung zu setzen.

## Akteure der Schlaganfallnachsorge und sektorenübergreifende Versorgung

Konzepte zur strukturierten Schlaganfallnachsorge beinhalten ein regelmäßiges Reassessment unter Berücksichtigung zahlreicher Gesichtspunkte – angefangen bei der Sekundärprophylaxe bis hin zum Management diverser nichtvaskulärer Komplikationen sowie der Heil- und Hilfsmittelversorgung [4]. Als Beispiel für ein solches strukturiertes Reassessment werden häufig die Canadian Stroke Best Practices angeführt [10]. Die fachlichen Herausforderungen der Schlaganfallnachsorge erfordern hierbei im besonderen Maße eine multidisziplinäre und sektorenübergreifende Behandlungsplanung und -umsetzung [9]. Der Schwerpunkt liegt hierbei im neurologischen Fachgebiet unter Einbindung verschiedener Fachdisziplinen und Berufsgruppen. Der Schlaganfall als Krankheitsbild ist fest im neurologischen Fachgebiet verortet und seit 2017 in der ICD 11 als neurologische Erkrankung anerkannt. Eine neurologische Betreuung sollte daher zentraler Bestandteil der Versorgung sein, insbesondere auch im sektorenübergreifenden Sinne von der Akutbehandlung über die Rehabilitation bis zur langfristigen bedarfsgerechten Nachsorge.

Aktuell konsultiert die überwiegende Mehrheit der Schlaganfallpatienten primär ihren Hausarzt nach Entlassung aus der stationären Versorgung [11]. Der Hausarzt ist erster Ansprechpartner des Patienten und sollte folglich eng in die ambulante Versorgung mit einbezogen werden. Ein enger Austausch zwischen betreuendem Neurologen und Hausarzt scheint daher für die Betreuung unerlässlich. Gleichermaßen müssen weitere Fachdisziplinen wie beispielsweise die Kardiologie und Psychiatrie eng in die Behandlung eingebunden werden. Die neurologische Behandlung ist hierbei integraler Bestandteil dieses multidisziplinären und interprofessionellen Nachsorgeteams.

Unter Berücksichtigung der gegenwärtigen Bündelung der fachlichen Expertise in der Schlaganfallversorgung im stationären Sektor erscheint es zudem sinnvoll, diesen im Rahmen einer sektorenübergreifenden Versorgung ebenfalls im Nachsorgebereich mit einzubeziehen und damit auch der dynamischen Entwicklung in der Schlaganfallmedizin Rechnung zu tragen.

## Unterschiede in regionalen Versorgungsstrukturen

Im landesweiten Vergleich unterscheiden sich die strukturellen Voraussetzungen zur Etablierung einer strukturierten Schlaganfallnachsorge. Dies betrifft auch die Versorgungsdichte mit Stroke-Units. Zudem müssen vorbestehende sektorenübergreifende Versorgungsstrukturen, wie sie beispielsweise im Rahmen der Integrierten Versorgung (IV) zur Anwendung kommen, berücksichtigt werden. Auch die individuelle Expertise im Rahmen der Schlaganfallnachsorge und Versorgungsdichte niedergelassener Neurologen und Hausärzte kann variieren. Ein Konzept zur Schlaganfallnachsorge muss solche regionalen Strukturunterschiede berücksichtigen.

Um eine flächendeckende Versorgung zu gewährleisten, ist es daher zielführend, sowohl auf vorbestehende stationäre Versorgungsstrukturen im Rahmen einer sektorenübergreifenden Versorgung als auch auf bestehende ambulante Behandlungsstrukturen zurückzugreifen. Im Vordergrund steht hierbei die Gewährleistung der ärztlichen Expertise unabhängig von deren Verortung. Dabei kann beispielsweise die Einführung einer Zusatzqualifikation im Bereich der Schlaganfallnachsorge dazu beitragen, die Nachsorge fächerübergreifend zu öffnen und somit unabhängig von regionalen Strukturunterschieden kurzfristig und flächendeckend zu sichern. Eine solche Umsetzung würde den zuständigen Landesärztekammern obliegen.

## Qualitätssicherung der Schlaganfallnachsorge

Eine hochwertige Schlaganfallnachsorge bedarf nicht nur der Formulierung entsprechender Qualitätskriterien, sondern vielmehr angemessener Instrumente zur Qualitätssicherung. Aktuelle Versorgungsstrukturen berücksichtigen diesen Aspekt nicht und bieten auch nicht die Voraussetzungen zur Umsetzung eines Qualitätssicherungsverfahrens. Besonderes Hindernis ist hierbei die Fragmentierung der Datenbestände in Deutschland, welche bereits der Identifikation von Patienten, die in einem solchen Verfahren Berücksichtigung finden würden, entgegensteht. Die Etablierung eines Nachsorgeregisters auf Basis ausgewählter Indikatoren unter Nutzung von Routine- sowie Outcomedaten wäre ein erster Schritt zur Ausarbeitung von Qualitätssicherungsstrategien. Wichtig ist die angemessene Finanzierung solcher Register auf Landes- oder Bundesebene. Darüber hinaus ist ein vereinfachter Zugang zu bereits jetzt kostenträgerseitig erhobenen Routinedaten durch Fachexperten notwendig. Von den Kostenträgern muss dahingehend eine intensivierte Zusammenarbeit gefordert werden. Zukünftige Versorgungsstrukturen müssen den Aspekt der Qualitätssicherung in besonderem Maße berücksichtigen.

Ein Weg zur Implementierung von Qualitätssicherungsstrategien könnte mittelfristig auch durch Zertifizierungsprozesse geebnet werden, wie sie im Rahmen der Akutversorgung durch die DSG und Stiftung Deutsche Schlaganfall-Hilfe (SDSH) bereits etabliert wurden [5]. Die Erfahrungen aus den Qualitätssicherungs- und insbesondere Zertifizierungsverfahren der Akutversorgung zeigen, dass die Akzeptanz und Umsetzung hierbei wesentlich an Anreize gekoppelt ist. Voraussetzung für eine Qualitätssicherung ist somit ein geeignetes Finanzierungsmodell.

## Zielgruppe der strukturierten Schlaganfallnachsorge

Da der Schlaganfall ein heterogenes Krankheitsbild mit unterschiedlichen Schweregraden darstellt, ergibt sich die Frage, ob verschiedene Subgruppen von Schlaganfallpatienten gleichermaßen von einer strukturierten Nachsorge profitieren und inwiefern spezielle Bedürfnisse berücksichtigt werden müssen. Versorgungsforschungsstudien fokussieren üblicherweise auf leicht betroffene Patienten, um die Studiendurchführung zu erleichtern bzw. sicherzustellen [12, 13]. Jedoch benötigen gerade schwer Betroffene eine intensive Nachsorge mit den notwendigen Therapien zum Erhalt bzw. der Wiederherstellung der Lebensqualität. Gleichermaßen können auch Patienten nach transienter ischämischer Attacke (TIA) im Rahmen einer gezielten Sekundärprävention von einer systematischen Schlaganfallnachsorge profitieren. Daher sollte das Angebot zur strukturierten Schlaganfallnachsorge nicht auf spezielle Subgruppen von Schlaganfallpatienten beschränkt werden. Zukünftig sollten die Anforderungen und der Nutzen einer systematischen Schlaganfallnachsorge in verschiedenen Subgruppen besser untersucht werden, um den divergierenden Bedürfnissen besser gerecht zu werden. Entsprechende Prioritäten wurden bereits 2018 im „Stroke Action Plan for Europe“ formuliert [8].

## Einbindung innovativer Technologien

Mit einer breiten Initiative, die u. a. das E‑Health-Gesetz im Jahr 2015, das Digitale-Versorgungs-Gesetz im Jahr 2019 und die Einführung der elektronischen Patientenakte im Jahr 2021 einschließt, unterstützt der Gesetzgeber den Einsatz digitaler Anwendungen im Gesundheitswesen [14]. Für die Schlaganfallnachsorge lassen sich hierdurch zahlreiche Vorteile ableiten. Beispielsweise erscheinen durch einen barrierefreien Austausch von Informationen intersektorale Schnittstellenprobleme lösbar und durch die Nutzung der digitalen Kommunikation werden Austausche zwischen den in der Nachsorge tätigen Akteuren und den Betroffenen vereinfacht. Innovative technische Lösungen können somit zukünftig einen wichtigen Beitrag zur Schlaganfallnachsorge leisten.

Darüber hinaus sind durch mobile patientennahe Technologien (mHealth) auch Vorteile in der Sekundärprophylaxe denkbar. So können mobile Endgeräte für Blutdruckmessungen unter Alltagsbedingungen – wie sie in den einschlägigen Leitlinien empfohlen werden [15] – eingesetzt werden oder mobile EKG-Geräte bei der frühzeitigen Detektion von Herzrhythmusstörungen helfen. Den zahlreichen theoretischen Vorteilen bei der Nutzung von E‑Health und mHealth steht u. a. mit der im Mai 2021 in Kraft getretenen Medizinprodukteverordnung und dem Medizinprodukterecht-Durchführungsgesetz jedoch ein komplexes Regelwerk im Entwicklungs- und Zulassungsprozess gegenüber, welches zu Unsicherheit führt und Innovation entgegensteht. Ob die mithilfe eines beschleunigten Verfahrens im Moment in großer Zahl entstehenden, meist auf einzelne Aspekte (z. B. körperliche Bewegung) begrenzten, digitalen Gesundheitsanwendungen (DiGA) den komplexen Nachsorgebedarfen gerecht werden, bleibt abzuwarten.

## Gesundheitspolitische und -ökonomische Aspekte

Die Förderung gleich mehrerer Projekte zur Optimierung der Schlaganfallnachsorge im Rahmen des Innovationsfonds des Gemeinsamen Bundesausschusses (G-BA) unterstreicht die zunehmende Bedeutung einer strukturierten Schlaganfallnachsorge in der gesundheitspolitischen Diskussion. Versorgungsforschungsstudien innerhalb und außerhalb Deutschlands zur Effektivität von Schlaganfallnachsorgeprogrammen erbrachten bisher gegenläufige Ergebnisse [12, 16, 17]. Hierbei muss berücksichtigt werden, dass sich die internationalen Erfahrungen und Ergebnisse aus der Versorgungsforschung aufgrund des spezifischen Aufbaus des deutschen Gesundheitssystems nur bedingt übertragen lassen. Mit Abschluss des STROKE-OWL(sektorübergreifend organisierte Versorgung komplexer chronischer Erkrankungen: Schlaganfall-Lotsen in Ostwestfalen-Lippe)-Projekts unter Führung der Deutschen Schlaganfall-Hilfe [18] steht nun jedoch die Diskussion der ersten Projektergebnisse aus der Förderung durch den Innovationsfonds im G‑BA an. Vielversprechend ist, dass dieses Projekt im Rahmen von IV-Verträgen mit den Leistungsträgern bis zur etwaigen Kostenübernahme im Rahmen der Regelversorgung nach Abschluss der Förderung durch den Innovationsfonds fortgeführt wird. Mit dem Projekt SANO zur strukturierten ambulanten Nachsorge nach einem Schlaganfall [13] sowie StroCare zur optimierten sektorenübergreifenden, koordinierten und evidenzbasierten Behandlung von Schlaganfallpatienten durch übergreifende Prozessverantwortung und patientenorientierte Ergebnisqualitätsmessung [19] ist zudem mit der Veröffentlichung der Ergebnisse der zwei weiteren Innovationsfondsprojekte zur Schlaganfallnachsorge Mitte 2022 bzw. 2023 zu rechnen. Teil der Evaluation dieser Projekte sind auch gesundheitsökonomische Analysen zur strukturierten Schlaganfallnachsorge.

Hierbei stehen den zusätzlichen Aufwendungen im Rahmen der strukturierten Schlaganfallnachsorge der gesundheitsökonomische Nutzen durch die Reduzierung von Abhängigkeit und Behinderung, den Aufwendungen für die Versorgung von Rezidivereignissen und Behandlung der Folgen nichtvaskulärer Sekundärkomplikationen sowie bestenfalls auch der Erhalt der Arbeitsfähigkeit gegenüber. Hervorzuheben ist, dass eine strukturierte Schlaganfallnachsorge keinesfalls kostenneutral im gesundheitsökonomischen Sinne sein muss, da den zusätzlichen Aufwendungen ein potenziell erheblicher Nutzen für die Patientinnen und Patienten gegenübersteht. Der Nachweis ist im Rahmen der gegenwärtigen Nachsorgeprojekte entsprechend zu erbringen. Die Einführung und Umsetzung einer strukturierten ambulanten Schlaganfallnachsorge scheint jedoch ohne eine ausreichende Finanzierung und hiermit verbundene eigene finanzielle Anreize nicht realisierbar.

## Diskussion spezifischer Versorgungs- und Finanzierungsmodelle

**Figure 1:**
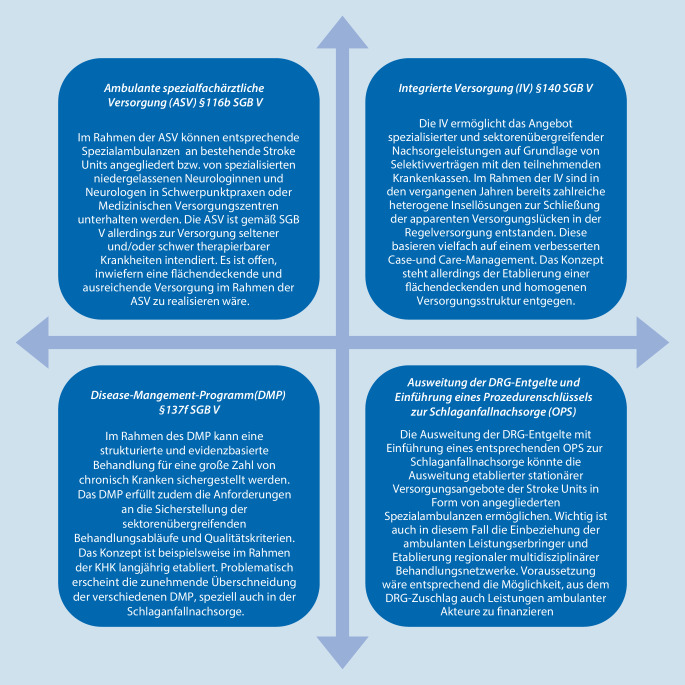

Versorgungsmodelle zur Schlaganfallnachsorge müssen eine strukturierte, berufsgruppen- und sektorenübergreifende Nachbehandlung in einem multidisziplinären Behandlungsnetzwerk sicherstellen. Eine strukturierte Schlaganfallnachsorge scheint dabei auf Grundlage bestehender Versorgungsrichtlinien in verschiedener Weise realisierbar (Abb. 1). So kann der Schlaganfall für die ambulante spezialfachärztliche Versorgung (ASV) nach § 116b SGB V geöffnet werden und damit bestehende Versorgungsstrukturen des stationären wie des ambulanten Sektors mit einzubeziehen. Eine Reevaluation des Katalogs hochspezialisierter Leistungen durch den G‑BA wäre in diesem Kontext sinnvoll. Das Konzept würde dabei gleichsam den besonderen Anforderungen an die Qualifikation des ärztlichen Personals, den diagnostischen und therapeutischen Leistungsumfang sowie den spezifischen Anforderungen an die multidisziplinäre Versorgung von Schlaganfallpatienten gerecht werden. Der Gesetzgeber hat die ASV durch die Einbeziehung schwer therapierbarer bzw. komplexer Erkrankungen in § 116b SGB V für eine Vielzahl chronischer Erkrankungen geöffnet. Dieses Kriterium erfüllt der Schlaganfall als chronische Erkrankung. Inwiefern mit der ASV eine flächendeckende Versorgung und hinreichende Einbeziehung ambulanter Versorgungsstrukturen realisierbar wäre, bleibt offen. Der hohe bürokratische Aufwand bei der Etablierung der ASV dürfte eine flächendeckende Versorgung eher erschweren. Ein entsprechendes Versorgungskonzept wäre zunächst durch den G‑BA festzulegen.

Hingegen stehen dem weiteren Ausbau bereits vorhandener sektorenübergreifender Versorgungsstrukturen im Rahmen der Integrierten Versorgung (IV) nach § 140 SGB V keine rechtlichen Hürden gegenüber. Seit Beginn des neuen Jahrtausends sind bereits zahlreiche regionale und überregionale Projekte auf Grundlage der IV entstanden [20]. Viele dieser Projekte wurden jedoch inzwischen wieder beendet. Eine flächendeckende Versorgung auf Basis der IV konnte bisher nicht etabliert werden. Besonderer Hinderungsgrund ist die einzelvertragliche Grundlage der IV mit regionalen und überregionalen Kostenträgern, welche das Konzept an das individuelle Engagement regionaler Akteure bindet. Das Konzept steht somit einer homogenen und flächendeckenden Versorgungsstruktur mit einheitlichen Qualitätsstandards entgegen. Die aktuelle Form der IV erscheint daher dauerhaft zur Sicherstellung der breiten Durchdringung und der Versorgungsqualität nach einem Schlaganfall ungeeignet.

**Figure 2:**
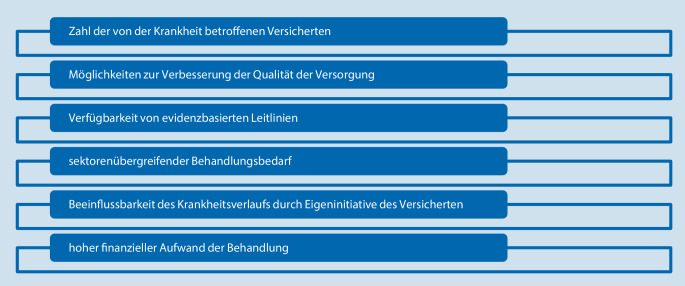

Die positiven Erfahrungen mit dem Disease-Management-Programm (DMP) zur koronaren Herzkrankheit (KHK) lassen aufgrund des vergleichbaren Risikoprofils ein analoges Modell für die ambulante Schlaganfallnachsorge attraktiv erscheinen. Im Rahmen des DMP können Behandlungsabläufe strukturiert und auch die sektorenübergreifende Behandlung gesichert werden. Teil des DMP sind insbesondere die Sicherstellung der evidenzbasierten Behandlung, die Umsetzung von Qualitätssicherungsmaßnahmen sowie die regelmäßige Schulung der Leistungserbringer. Im Rahmen des DMP soll die Behandlung chronisch Erkrankter langfristig gesichert und somit Komplikationen und Folgeschäden vorgebeugt werden. Bezogen auf den Schlaganfall kann hierdurch nicht nur die leitliniengerechte Behandlung der vaskulären Risikofaktoren und nichtvaskulären Komplikationen, sondern auch die angemessene Heil- und Hilfsmittelversorgung sichergestellt werden. Die wesentlichen Voraussetzungen für die Entwicklung eines DMP gemäß SGB V sind in Abb. 2 zusammengefasst und auf die Schlaganfallnachsorge anwendbar. Das DMP wurde in der Vergangenheit entsprechend bereits vielfach für den Schlaganfall als Versorgungsmodell diskutiert [21].

Die Bewertung des therapeutischen oder diagnostischen Nutzens einer Maßnahme bzw. DMP im G‑BA ist im besonderen Maße an die aktuelle wissenschaftliche Evidenz gebunden. Es ist anzunehmen, dass die bisher fehlende Evidenz zum Nutzen der strukturierten Schlaganfallnachsorge der Aufnahme und Entwicklung eines entsprechenden DMP im Wege stand. Die zu erwartende umfangreiche Evidenz in diesem Sektor lässt somit eine Neubewertung des G‑BA und letztlich eine Entwicklung eines DMP Schlaganfall wieder möglich erscheinen. Eine Voraussetzung ist jedoch die aktuell noch ausstehende Definition entsprechender Qualitätsindikatoren. In jedem Fall müsste das DMP auch für Neurologen als koordinierende Ärzte geöffnet werden, um den zuvor genannten Argumenten sowie Erfahrungen aus den Innovationsfondsprojekten Rechnung zu tragen. Ein vergleichbares Konzept ist bereits im Rahmen des DMP Diabetes mellitus Typ 2 umgesetzt [22]. Nicht verschwiegen werden darf, dass es mit zunehmender Zahl an DMP vermehrt zu Überschneidungen bei diesen kommt und Patienten folglich an mehreren DMP teilnehmen. Diese Problematik ist auch für den Schlaganfall und dessen Überschneidung mit anderen kardiovaskulären Erkrankungen, deren Risikofaktoren und nichtvaskulären Komplikationen (z. B. DMP zu KHK, Diabetes mellitus, COPD und Depression) zu beachten. Gerade im Kontext der Schlaganfallnachsorge erscheint dies nicht unproblematisch und zeigt die Notwendigkeit individueller Versorgungskonzepte mit einer angepassten und leistungsgerechten Vergütung auf.

Auf die Vorteile der Einbindung bestehender stationärer Versorgungsstrukturen in die ambulante Schlaganfallnachsorge wurde eingangs bereits hingewiesen. Als ein attraktives Model kann daher die transsektorale Ausweitung der bestehenden stationären Versorgungsangebote der Stroke-Units auf Basis einer leistungsbezogenen Ausweitung der DRG-Entgelte und Etablierung eines eigenen Prozedurenschlüssels (OPS) zur Schlaganfallnachsorge diskutiert werden. Eine entsprechende OPS könnte dahingehend definiert werden, dass sie die vorangehend diskutierten Qualitätsmerkmale einer strukturierten und berufsgruppenübergreifenden Nachbehandlung im Rahmen eines multidisziplinären Behandlungsnetzwerkes umfasst. Voraussetzung für ein Gelingen wäre die Möglichkeit, zusätzlich zum DRG-Zuschlag noch Mittel für Leistungen ambulant tätiger, in das Konzept integrierter Akteure zu generieren.

Ein realistisches Ziel ist die Ausweitung und Vergütung transsektoraler Behandlungsangebote der Stroke-Units mit Etablierung angegliederter multidisziplinärer regionaler Behandlungsnetzwerke. Ein entscheidendes Merkmal ist dabei, dass ein solches Versorgungsmodell ambulante Leistungserbringer mit einbezieht und regionale Versorgungsstrukturen berücksichtigt. Hierfür müssen angemessene finanzielle Anreize für die Leistungserbringer vorgehalten und Finanzierungsmodelle entwickelt werden. Langfristig könnten sektorenübergreifende Behandlungsangebote auch ein essenzieller Bestandteil von Zertifizierungsprozessen sein.

## Fazit

Die Diskussion neuer Versorgungsformen zur Schlaganfallnachsorge erfährt gegenwärtig ein neues Momentum und eröffnet Perspektiven für eine tatsächliche Verbesserung der aktuell noch unzureichenden Versorgungslösung. Die Einführung einer flächendeckenden strukturierten ambulanten Schlaganfallnachsorge wird ohne eine ausreichende Finanzierung und hiermit verbundene eigene finanzielle Anreize nicht realisierbar sein. Die Etablierung und Finanzierung ist hierbei auf Grundlage verschiedener Modelle vorstellbar. Eckpunkte sind eine ausreichend flexible Ausgestaltung zur flächendeckenden Gewährleistung evidenzbasierter Behandlungsstandards, hiermit verbundene Instrumente zur Qualitätssicherung und eine multidisziplinäre und transsektorale Versorgung in einem regionalen Versorgungsnetzwerk. Die Vor- und Nachteile klassischer Versorgungs- und Finanzierungsmodelle sind vor dem Hintergrund der zugrunde liegenden medizinischen Anforderungen und gegenwärtigen strukturellen Voraussetzungen kritisch gegeneinander abzuwägen. Die anstehende Evaluation gleich mehrerer Innovationsfondsprojekte wird dabei eine ernsthafte und evidenzbasierte Diskussion über die Zukunft der Schlaganfallnachsorge ermöglichen. Dies bezieht auch neu konzipierte, transsektoral ausgerichtete Versorgungsformen wie eine Ausweitung der Angebote der bereits flächendeckend vorhandenen Stroke-Units in den ambulanten Sektor mit ein, wofür jedoch ein entsprechendes Finanzierungsmodell die bisherige Trennung des stationären und ambulanten Sektors überwinden müsste.
